# The impact of COVID-19 on blood supply–demand balance and the association between group donations and economic performance: evidence from Zhejiang, China

**DOI:** 10.3389/fpubh.2026.1739767

**Published:** 2026-02-16

**Authors:** Guan Wang, Xiaoying Pu, Renzo Jose Carlos Calderon Anyosa, Yaming Gu

**Affiliations:** 1 Institute of Health Policy, Management and Evaluation, University of Toronto, Toronto, ON, Canada; 2 School of Basic Medical Sciences, Hangzhou Normal University, Hangzhou, China; 3 School of Public Health, Hangzhou Medical College, Hangzhou, China

**Keywords:** blood supply and demand, China, COVID-19, economic performance, group donations

## Abstract

**Background:**

The COVID-19 pandemic significantly disrupted the balance between blood supply and demand; however, limited evidence reveals how this imbalance emerged, particularly across specific blood types. Changes in donation patterns during the pandemic and their association with the local economy also remain underexplored.

**Methods:**

Using provincial-level data from 2014 to 2023, we used an interrupted time series model to analyze the trends in blood supply, blood demand, and group donations caused by the pandemic. Generalized least squares and distributed lag models were applied to examine the relationship between group donations and local economic performance.

**Results:**

The pandemic led to an immediate decline in the supply-to-demand ratio for blood, with types A and O most severely affected. Group donations were positively associated with local economic performance, although COVID-19 attenuated this relationship.

**Conclusion:**

Tailored strategies are needed for blood types in high demand. Supporting group donations may help promote economic resilience during public health crises.

## Introduction

In 2020, COVID-19 spread rapidly across the globe, disrupting nearly every aspect of public life, with the healthcare system bearing the greatest burden (1). Among the affected sectors, blood donation and transfusion services were the most affected, as they are highly sensitive to fluctuations in supply and demand. Zhejiang province, one of the most developed regions in China, responded quickly by implementing effective strategies to minimize infection rates and alleviate pressure on the local healthcare system (2). Working in partnership with local businesses, the provincial government introduced innovative tools, such as the health code system, to help with individual health management, including tracking epidemic history, and to prevent further outbreaks (2, 3). Although the pandemic was eventually contained within a relatively short period in Zhejiang, it inevitably changed individual behaviors—individuals initially delayed seeking healthcare, leading to a subsequent surge in demand for medical needs as they attempted to address previously postponed needs (4).

Meanwhile, the pandemic also caused broad consequences beyond its immediate health impacts (5). Movement restrictions and reduced mobility disrupted individuals’ regular access to blood donation centers. Blood centers also introduced additional donor screening and deferral criteria to protect donors and safeguard the blood supply, which further decreased the number of eligible donors (6). Together, these disruptions may lead to short-term declines in blood collections and, in turn, increase the risk of supply shortages and creating downstream challenges for hospitals and patients reliant on transfusions. This public health crisis, therefore, exposed the inherent vulnerability of the blood supply chain, revealing how quickly external shocks may destabilize a system that depends on continuous donor participation. The severity of disruption varied by blood type, with blood type O disproportionately affected the most due to higher clinical demand (7). An adequate blood supply ensures that patients receive timely transfusions, which helps prevent treatment delays and reduces time away from work. Previous studies also show that a well-functioning blood supply–demand system not only helps prevent medical complications but also reduces long-term healthcare costs, thereby supporting both individual well-being and the overall performance of the healthcare system (8).

In China, the blood donation system primarily comes through two channels: individual donations and group donations (9). Individual donations typically involve people who spontaneously visit blood donation sites, often motivated by personal initiative or public appeals. By contrast, group donations are usually organized by community organizations, schools, or workplaces, with blood drives scheduled at specific times (10). To facilitate group donations, blood centers often provide on-site collection services, although this generally requires mobilizing a minimum number of donors to be cost-effective (11). This approach is convenient and efficient for maintaining a stable blood supply. As a complement to individual donations, group donations embody collective action and social solidarity, particularly in the Chinese context, where community-oriented initiatives have strong cultural and institutional support.

During the COVID-19 pandemic, the blood collection-to-supply process could be disrupted through multiple channels. In general, lockdowns and reduced mobility decreased donor attendance and disrupted blood collection. In addition, blood centers implemented additional donor screening and deferral criteria to protect donors and maintain the safety of the blood supply, which could further reduce the number of eligible donors. Together, these disruptions may lead to short-term declines in collections and, in turn, reduce the stability of the blood supply. In addition to affecting blood donations, COVID-19 also has negative impacts on the economy (12). This disruption manifested in severe shocks to the retail, service, and manufacturing industries caused by lockdowns (13). These economic disturbances are intertwined with challenges within the health system, as resource shortages—including those in the blood supply chain—undermined labor productivity and further exacerbated the economic recession. Given this dual burden on both the economy and health system, understanding the correlations between COVID-19, the blood system, and economic performance becomes particularly important. Previous studies have explored the impact of COVID-19 on either blood supply or demand (14, 15), but very few have investigated both aspects simultaneously. Additionally, these studies have not considered its impact on group donations. At the same time, although the correlation between economic development and human health has been widely studied, little is known about the specific role of group donations in supporting economic resilience—especially in the context of COVID-19, when the individual donation channel was severely disrupted. Addressing these gaps is important for both public health and policy planning. From a healthcare perspective, understanding variations in supply and demand by blood type can inform targeted strategies to secure vulnerable blood groups. From an economic perspective, clarifying how group donations contribute to economic performance provides valuable evidence for mobilizing community-based initiatives in future crises. Using provincial-level data from Zhejiang, this study primarily aimed to answer two key questions:

1. How did COVID-19 affect the balance between blood supply and demand in Zhejiang province, both overall and by blood type?
2. To what extent did those changes in blood supply and demand influence local economic performance in the COVID-19 context, and what role did group donations play in supporting the economy?
  We also included interaction terms between the blood variables and the COVID-19 indicator to quantify how COVID-19 modified their associations with the outcome.

## Methods

### Dataset and variables

We obtained data from the provincial blood center, including weekly-level total blood supply and demand, and their breakdowns by blood types A, B, O, and AB. The dataset also details the group donations and total collection volume between 1 January 2014 and 15 December 2023. All blood collection volumes were expressed in units (U) (see Supplementary material). To further assess the impact of blood supply, demand, and group donations on the local economy, we linked our data to the Provincial Bureau of Statistics from 2014 to 2023. We included gross regional product (GRP) as a proxy for economic performance, as it reflects local productivity, living standards, and income levels. Population size and healthcare workers per 1,000 population were also included in further analyses (see Supplementary material).

### Statistical analysis

Descriptive statistics were first used to summarize blood supply, blood demand, supply-to-demand ratios, group donations, and health resource indicators before and after the onset of COVID-19. Means and standard deviations (SD) were calculated for the pre- and post-pandemic periods. Paired t-tests were performed to examine the differences in mean values between the two periods, and corresponding *p*-values were reported to assess whether the observed changes were statistically significant, using a threshold of *p* < 0.05. These results are presented in Table 1.

**Table 1:** Description analysis of the main variables.

Main variables	Before COVID (mean ± SD)	After COVID (mean ± SD)	Change in percentage (%)	*p*-values
Blood supply	3,827.381 ± 587.431	4,589.472 ± 923.091	19.912	0.000*
Blood demand	3,910.330 ± 558.579	4,910.791 ± 663.156	25.585	0.000*
Supply-to-demand	0.981 ± 0.083	0.930 ± 0.102	−5.205	0.000*
Supply-to-demand blood type A	0.960 ± 0.117	0.899 ± 0.132	−6.336	0.000*
Supply-to-demand blood type B	1.031 ± 0.229	0.976 ± 0.130	−5.279	0.001*
Supply-to-demand blood type O	0.970 ± 0.096	0.906 ± 0.116	−6.567	0.000*
Supply-to-demand blood type AB	1.068 ± 0.432	1.025 ± 0.248	−4.033	0.151
Group donations	1,610.092 ± 905.767	2,004.426 ± 1,222.077	24.491	0.000*
Total collections	3,647.937 ± 969.584	4,350.965 ± 1,322.556	19.272	0.000*
Group donations proportion	0.415 ± 0.160	0.427 ± 0.163	2.921	0.408
Healthcare workers per 1,000 population	7.898 ± 0.7104	9.160 ± 0.589	15.974	0.000*

*p < 0.01.

### COVID-19 and blood supply/demand, group donation proportion

**Figure 1:**
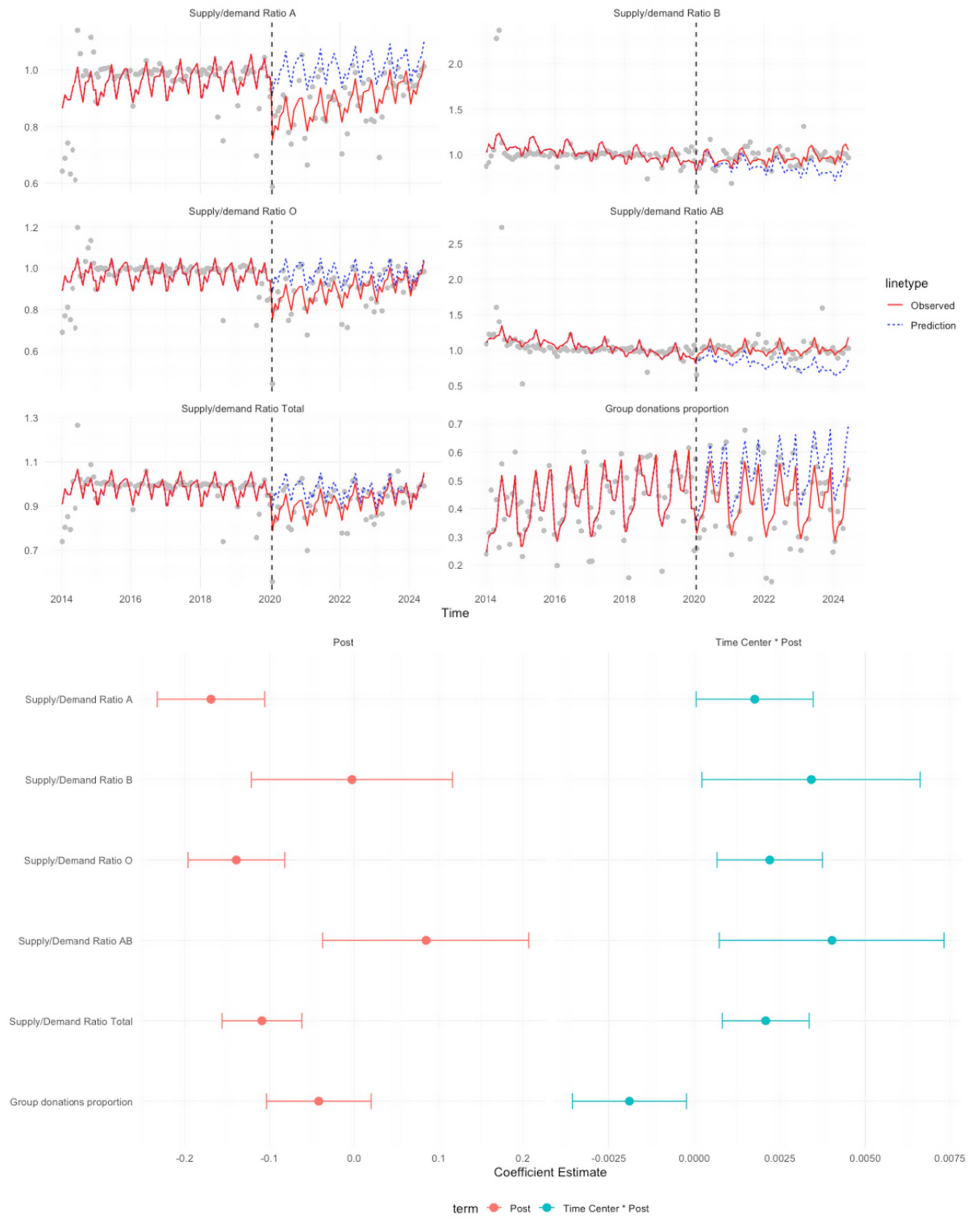
ITS estimates of COVID-19-associated changes in the blood supply to demand ratio and group donation proportion.

**Figure 2:**
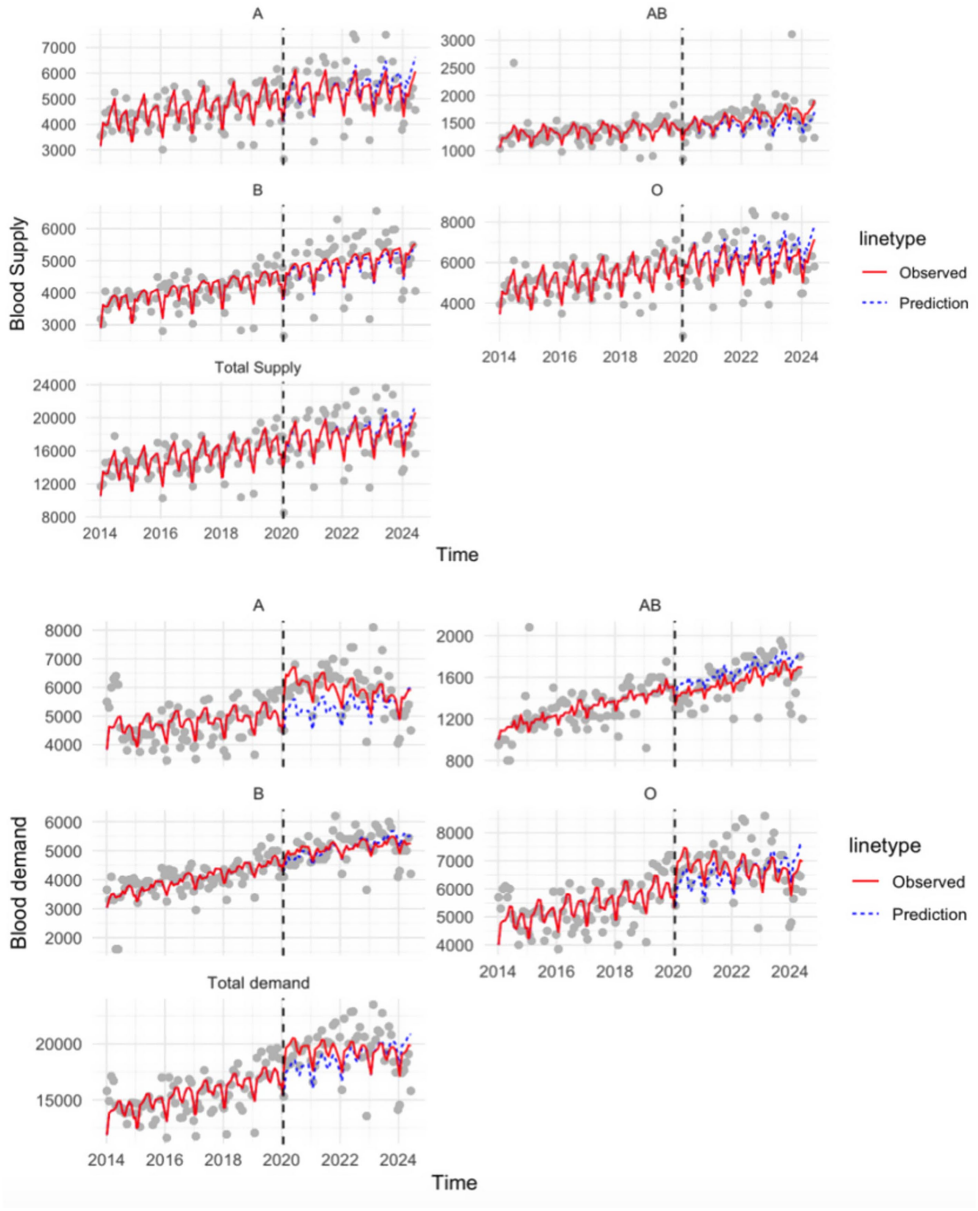
ITS estimates of COVID-19-associated changes in blood supply and demand by blood type.

For this section, we applied the interrupted time series (ITS) method. 21 January 2020 was taken as the onset of the COVID-19 outbreak in Zhejiang (16). ITS is a quasi-experimental approach that allows us to assess the impact of an intervention or event when randomization is not feasible (17, 18). By modeling the data from the period before the event, we can extrapolate what the trend would have been in the absence of the event, yielding a counterfactual scenario. The difference between this projected trend and the actual data observed after the event provides an estimate of immediate or long-term impacts (19). Our outcomes of interest included the blood supply to demand ratio and the proportion of group donations in total collection. We also conducted analyses of blood supply and demand separately as secondary outcomes to examine their independent trends over time.

$$Y_{t}=\beta_{0}+\beta_{1}T_{t}+\beta_{2}\text{COVI}D_{t}+\beta_{3}\text{COVI}D_{t}*T_{t}+\in_{t}$$

where $Y_{t}$ represents the outcome variable measured at each time point t. $T_{t}$ captures the time since the study began, while $\text{COVI}D_{t}$ is a dummy variable denoting the occurrence of COVID-19. The interaction term $\text{COVI}D_{t}*T_{t}$ enables us to assess changes over time in response to the event. We grouped the weekly data on a monthly level and accounted for seasonality by including month-year fixed effects. In this model, $\beta_{0}$ represents the initial level of the outcome variable at the start of the study, and $\beta_{1}$ denotes the slope of the outcome variables before the event happened. $\beta_{2}$ denotes the immediate change in the level of outcome due to the event (compared with the counterfactual). $\beta_{3}$ quantifies the difference between the pre- and post-event trends of the outcome, offering insight into changes in the trajectory following the event. Statistical inference focused on the estimated coefficients $\beta_{2}$ and $\beta_{3}$ ; corresponding *p*-values and 95% confidence intervals (CI) were calculated using model standard errors and are reported in Figure 1. ITS estimates of blood supply and demand by blood type are presented in Figure 2.

Given the context, we expected our $\beta_{2}$ to be negative, reflecting the immediate adverse impact of COVID-19 on blood supply/demand and group donation proportion. In contrast, $\beta_{3}$ could be either negative or positive relative to $\beta_{1}$ (the pre-COVID trend), indicating a long-term downward trajectory due to persistent negative effects of the pandemic, or an upward slope reflecting recovery over time.

### Blood supply, blood demand, group donations, and economic performance

To explore the association between blood variables and local economic performance, we used a generalized least square (GLS) model, as this approach addresses the autocorrelation and heteroskedasticity of time-series data. We first used the augmented Dickey–Fuller (ADF) test to assess the stationarity of our variables. We then performed differencing as needed to ensure the variables of interest were stationary. The model specification was expressed as:

$$\begin{array}{l} \mathrm{GRP}\;\mathrm{per}\;\text{capit}a^{\text{Quarter}}=\beta_{0}+\beta_{1}{\left(\text{Blood Supply}\right)}_{t}\\ +\beta_{2}{\left(\text{Group donations}\right)}_{t}+\beta_{3}X_{t}+\beta_{4}\text{COVI}D_{t}+\in_{t} \end{array}$$

Here, GRPpercapita represented the gross regional product divided by population size, and deflated using the quarterly Consumer Price Index, with 2014 specified as the base period to adjust for monetary inflation. Independent variables included blood supply, group donations, blood demand, total blood collection, healthcare workers per 1,000 population, and the index of COVID occurrence. $\text{COVI}D_{t}$ was a binary variable (coded as 0 for pre-COVID periods and 1 otherwise). $\beta_{1}$ − $\beta_{4}$ denoted regression coefficients representing the association between each predictor and GRPpercapita . All right-hand-side variables were grouped at the quarterly level to align with the granularity of our outcome variables.

Since GLS only captured the association between blood-related variables and economic performance, to further explore the potential causal effects, we included lagged variables and examined their effects on the current time period outcome by using a distributed lag model (DLM). DLM captures the delayed effects of independent variables over multiple time periods, enabling the investigation of whether these effects persist or dissipate over time.

$$\begin{array}{l} \mathrm{GRP}\;\mathrm{per}\;\text{capit}a^{\text{Quarter}}=\beta_{0}+\sum_{i=0}^{2}\beta_{1i}{\left(\text{Blood supply}\right)}_{t-i}\\ +\sum_{i=0}^{2}\beta_{2i}{\left(\text{Group donations}\right)}_{t-i}+\sum_{i=0}^{2}\beta_{3i}X_{t-i}+\beta_{4}\text{COVI}D_{t}+\in_{t} \end{array}$$

where $\beta_{1i}$ , $\beta_{2i}$ , and $\beta_{3i}$ were coefficients for the current ( i=0 ) and lagged ( i=1,2 ) effects of blood supply, group donations, and other predictors, respectively. t−i was the value from previous quarters, where i denoted the lag length. Lagged terms for each variable allowed us to observe whether changes in previous quarters influenced the current outcome. The model incorporated up to two lags ( i=1,2 ) to identify the most relevant prior periods that could affect the current economic performance. By including lagged terms, we were able to reveal whether blood supply, demand, and group donations have a sustained or merely temporary impact on the local economy. Statistical significance of GLS and DLM regression coefficients was assessed using two-sided hypothesis tests; all statistical analyses were conducted at the 5% significance level. The results of the analyses are presented in Tables 2, 3.

**Table 2:** Impacts of blood supply, demand, and group donations on the economy.

Predictors	Model 1		Model 2		Model 3	
	Coefficients (SE)	*p*-value	Coefficients (SE)	*p*-value	Coefficients (SE)	*p*-value
Blood supply	0.132 (0.151)	0.386	0.096 (0.309)	0.759	0.277 (0.365)	0.456
Blood demand	−0.403 (0.114)	0.0013*	−0.470 (0.179)	0.0140*	−0.555 (0.222)	0.0205*
Group donations	0.214 (0.064)	0.0020*	0.243 (0.108)	0.0326*	0.164 (0.134)	0.233
Total collected	−0.023 (0.109)	0.834	0.090 (0.248)	0.720	0.023 (0.268)	0.933
Blood supply (lag1)			0.097 (0.266)	0.720	0.275 (0.474)	0.568
Blood demand (lag1)			−0.088 (0.170)	0.608	−0.283 (0.245)	0.261
Group donations (lag1)			0.074 (0.239)	0.941	0.021 (0.175)	0.905
Total collected (lag1)			0.018 (0.239)	0.941	0.064 (0.351)	0.856
Blood supply (lag2)					0.160 (0.310)	0.611
Blood demand (lag2)					−0.206 (0.187)	0.282
Group donations (lag2)					0.001 (0.129)	0.993
Total collected (lag2)					0.098 (0.258)	0.706
Healthcare workers per 1,000 population	641.990 (793.382)	0.424	926.492 (946.134)	0.336	757.333 (1,066.326)	0.485
COVID-19	−1,124.106 (1,396.632)	0.427	−1,385.212 (1,546.678)	0.378	−1,321.418 (1,641.820)	0.429

Model 1 was the baseline model; Model 2 included one lag, and Model 3 included up to two lags. * *p* <0.01.

**Table 3:** Impacts of inducing interaction terms with COVID-19 on the economy.

Predictors	Model 1		Model 2		Model 3	
	Coefficients (SE)	*p*-value	Coefficients (SE)	*p*-value	Coefficients (SE)	*p*-value
Blood supply	0.022 (0.215)	0.919	0.399 (0.362)	0.284	−0.00233 (0.939)	0.998
Blood supply interacted with COVID-19	0.018 (0.256)	0.943	−0.391 (0.475)	0.420	0.744 (0.948)	0.450
Blood demand	−0.504 (0.166)	0.0050*	−0.809 (0.279)	0.0092*	−0.474 (0.419)	0.284
Blood demand interacted with COVID-19	0.148 (0.193)	0.448	0.308 (0.337)	0.373	−0.490 (0.458)	0.310
Group donations	0.790 (0.136)	0.000*	0.892 (0.201)	0.0003*	0.944 (0.246)	0.0033*
Group donations interacted with COVID-19	−0.622 (0.146)	0.0002*	−0.640 (0.207)	0.0060*	−0.870 (0.279)	0.0109*
Total collection	0.124 (0.229)	0.594	0.078 (0.429)	0.858	0.134 (0.669)	0.845
Total collection interacted with COVID	−0.150 (0.244)	0.543	0.103 (0.476)	0.831	0.741 (0.709)	0.320
Healthcare workers per 1,000 population	898.978 (593.276)	0.141	617.485 (706.891)	0.393	17.719 (712.087)	0.981
COVID-19	−1,155.694 (1,021.179)	0.267	−796.198 (1,106.485)	0.481	−206.464 (911.804)	0.825

Model 1 included the interaction term with COVID-19; Model 2 additionally controlled for one lag; Model 3 controlled for up to two lags. * *p* <0.01.

## Results

### Descriptive analysis

From Table 1, we observed significant changes in blood supply and demand by blood type before and after COVID-19. The average increase in blood demand exceeded the average change in blood supply, leading to a decline in the overall supply-to-demand ratio—a decrease of 5.2% (*p* < 0.01). This decline was consistent across the majority of blood types, with blood type A experiencing the largest decrease, from 0.960 to 0.899 (*p* < 0.01). Blood types O and B also showed notable declines in their supply-to-demand ratios, decreasing by 6.57 and 5.28%, respectively. In contrast, the supply-to-demand ratio for blood type AB exhibited a smaller change, reducing by only 4.03% (*p* = 0.151). Meanwhile, both group donations and total collections had a significant increase after the pandemic, with increases of 24.5 and 19.3%, respectively.

### Impact of COVID-19 on the blood supply/demand ratio and group donation proportion

Figure 1 presents the ITS analysis of supply-to-demand ratios by blood type and group donation proportion. The vertical dashed line marked the onset of COVID-19 in Zhejiang. In the upper panel, the overall supply-to-demand ratio exhibited a noticeable downward shift (*p* < 0.001). Blood types A and O experienced a more significant decline following the onset of the pandemic. The observed divergence from predicted counterfactual trends indicated a strong immediate impact. Unlike blood type O, which showed signs of recovery over time, blood type A maintained a lower ratio throughout the period. In contrast, trends for blood types B and AB suggested that the pandemic had less immediate and long-lasting effects on their supply-to-demand ratios. The forest plot showed that although the CIs for all blood types did not include zero, their coefficients were only marginally different from 0 and were accompanied by wide CIs, indicating that post-COVID-19 trends in the blood supply-to-demand remained relatively stable over time.

Figure 2 shows that both blood supply and demand exhibited upward trends across all blood types. The total blood supply had a relatively steady and consistent increase over time, with less obvious disruptive changes following the pandemic. Blood types A, B, O, and AB all showed similar gradual growth patterns. Compared to blood supply, blood demand displayed more distinct post-pandemic changes. We observed a significant increase in blood demand for blood types A and O. This sudden surge in demand caused a temporary imbalance in their supply-to-demand ratios. In contrast, demand for type B remained relatively stable during COVID-19, while type AB experienced a short-term decline. The overall upward trend in blood demand was primarily driven by substantial increases in the demand for blood types A and O.

### Impact of blood supply, demand, and group donations on local economic performance

To analyze the relationships between blood supply, demand, and group donations on local economic performance, we used GLS and DLM approaches. We performed first-order differencing of our variables of interest for stationarity and included both raw and lagged variables in the models. We also included interaction terms between COVID-19 and the variables to examine whether the pandemic moderated their relationships with local economic performance. The results are presented in Tables 2, 3.

Table 2 presents the impact of blood supply, demand, and group donations on the local economy, as measured by GRP per capita. The results indicated that a one-unit increase in group donations was associated with a 0.214 Chinese currency increase in GRP per capita (*p* = 0.0020). In contrast, a one-unit increase in blood demand corresponded to a 0.403 Chinese currency decrease in GRP per capita (*p* = 0.0013). When accounting for a one-quarter lag in the blood-related variables, the effects remained consistent but changed slightly in magnitude. Specifically, the effect of group donations on GRP per capita increased to 0.243 Chinese currency (*p* = 0.0326), while the effect of blood demand became more pronounced, with GRP per capita decreasing by 0.470 Chinese currency (*p* = 0.0140). These findings underscore the dual importance of maintaining an adequate blood supply to meet individual blood demand and of using group donations as a potential channel to promote economic resilience, especially during the pandemic.

Table 3 illustrates the moderating effects of COVID-19 on group donations. After accounting for the interaction term, group donations still showed a strong positive effect, increasing GRP per capita by 0.790 Chinese currency per unit increase (*p* < 0.01). This effect remained strong and statistically significant even after accounting for one- and two-quarter lags. However, the interaction terms indicated that the onset of COVID-19 significantly attenuated this positive effect. Specifically, the estimated impact of group donations declined by 0.622 Chinese currency (*p* = 0.0002). This moderating effect also persisted after incorporating lagged variables, further reducing the impact by 0.640 with a one-quarter lag (*p* = 0.0060) and by 0.869 with a two-quarter lag (*p* = 0.0109). These findings implied that the pandemic significantly weakened the positive impact of group donations on the local economic performance.

## Discussion

The COVID-19 pandemic exposed significant vulnerabilities in blood supply chains (20, 21). Our analysis also revealed a notable decline in the overall supply-to-demand ratio during the pandemic, with blood types A and O being particularly affected. While previous studies attributed blood supply challenges during the pandemic to lockdowns and movement restrictions (22), our study did not find a significant decline in blood supply. Instead, the sharp increase in demand for type A and type O indicated that targeted strategies should be developed for specific blood types. The decline in blood demand for type AB after the COVID-19 outbreak may reflect pandemic-related changes in healthcare utilization and hospital case mix, particularly during the early outbreak period. One possible explanation is that the type AB group had been disproportionately affected during the onset of the pandemic. Some studies have reported that group AB was positively associated with a higher mortality risk compared to the other blood types (23, 24). In addition, hospitals implemented patient blood management strategies during periods of constrained supply, which reduced transfusion utilization (25). Since AB recipients represent a relatively small share of the population, AB demand is more sensitive to short-term fluctuations and may show larger relative declines in aggregated time-series data. Blood type O had high demand during the pandemic due to its unique universal compatibility with transfusions. When supply fails to meet its elevated demand, it can place additional strain on healthcare systems. Blood type A was also heavily impacted. One possible reason is that, although type A is relatively common, it is less prevalent than type O in the local population (26), which might limit the available donor pool compared with the most prevalent blood type. Additionally, previous studies identified blood type A as a potential risk factor for COVID-19 infection (27, 28). Individuals with blood type A face a higher risk of contracting COVID-19, which could indirectly affect blood availability during outbreak periods. For example, illness or quarantine could have reduced participation among type A donors. This might further increase the clinical need for transfusions (27). Given the relatively stable blood types B and AB, it is important to implement tailored strategies to protect the supply of blood types that are more vulnerable during public health crises.

We did not find a long-term negative impact on the blood supply-to-demand ratio, which could be attributed to proactive measures implemented by blood centers, including extending the operating hours for donation sites and deploying mobile blood donation vehicles to improve accessibility for donors (29). Meanwhile, local organizations and communities coordinated group donations to meet the high demands for specific blood types (30). We found the pattern of blood donations shifted under the pandemic, with group donations taking up a greater proportion of the total blood collections, which serves as an alternative to traditional individual voluntary donations (31). Before the pandemic, individual donations made up a significant share of total blood collections. The pandemic somehow accelerated the transition to group donations. Local governments and private companies worked closely with blood centers to organize blood drives. Group donation offers the advantage of allowing collections to be scheduled in advance and enabling an efficient collection of larger blood volumes within a limited period (9, 11). This collaborative effort offsets the decline in individual voluntary donations, playing a crucial role in sustaining healthcare resources during the crisis. Other than that, the local government implemented demand-management measures, including promoting autologous transfusions, postponing elective surgeries, and adopting restrictive transfusion strategies with low hemoglobin thresholds. Collectively, these measures reduced non-essential blood use, helped preserve blood inventories, and maintained blood availability for emergency clinical needs (32).

Our analysis found that higher blood demand was negatively correlated with local economic performance. This is not surprising: even when blood supply remains within a normal range, once demand exceeds supply, the balance is broken, and shortages emerge, leading to adverse downstream influences. These imbalances disrupt timely medical care, prolong recovery times, and increase absenteeism, all of which negatively impact economic output. In contrast, the results revealed that an increase in group donations was positively associated with improvements in GRP per capita. Adequate blood donations were found to help reduce the number of individuals on sick leave (33). While this association did not establish causality due to insignificant lag variables, it nonetheless reflects the potential benefits of strengthening the healthcare system through timely blood transfusions. Since it ensures patients receive appropriate clinical treatment, thereby aiding their recovery and reducing workforce disruptions, this ultimately improves individuals’ work productivity (33). Furthermore, group donations facilitated the rapid collection of blood types in shortage, enabling an effective response to clinical needs (34). Community mobilization and collective efforts served as effective avenues for group donations in crisis management, helping to reduce blood shortages and ensure sufficient collection, further contributing to economic resilience during a public crisis. Importantly, our results do not imply group donation should replace individual donation in routine settings; rather, it complements individual donation and may be particularly valuable during emergency situations. Although the interaction term between COVID-19 and group donations was significantly negative—likely due to travel restrictions and logistical challenges faced by blood centers (35)—maintaining or expanding group donations can still be beneficial, since it helps buffer external shocks to the blood supply chain.

Our study focuses on Zhejiang Province, and therefore, the estimated results should be interpreted as province-specific. However, we believe that the main mechanisms identified in our analysis are likely relevant to broader policy planning. First, the ABO distribution in Zhejiang is broadly comparable to the national level, with type O, type A, and type B being the most prevalent blood types (26). This implies that supply–demand imbalances for common blood types are policy relevant beyond Zhejiang. Second, our findings highlight the role of group donation as a complementary channel to individual donations, especially during a public health crisis. We also notice existing regional heterogeneity referring to blood demand and supply in both ordinary and pandemic periods. Before COVID-19, blood supply and demand differed across regions due to variations in population structure, i.e., social and cultural ideas affecting donation willingness, and differences in blood collection infrastructure and management capacity (36, 37). Demand can also vary because of differences in ABO distributions, local healthcare service capacity, i.e., areas with more intensive hospital services may have higher clinical needs, and broader socioeconomic conditions (38). During COVID-19, heterogeneity likely increased further because lockdown stringency and duration differed across regions, donation opportunities, and logistics challenges (39, 40). While our results are context-specific, they provide policy-relevant evidence that can serve as a useful reference for other provinces and for settings with similar blood collection systems.

Our study is not without limitations. First, in our DLM model, it is important to recognize the insignificance of lagged variables when making causal inferences regarding the effect of group donations on economic performance, where the positive coefficient for group donations only indicated a positive correlation between these two variables. Second, our GRP per capita data were available only at a quarterly level, which limited our ability to capture more granular fluctuations that may occur at higher frequencies. This lower resolution may have smoothed over important variations in economic performance that could have been detected with more frequent data. Third, our outcome of interest may be influenced by other variables correlated with either blood supply, demand, or group donations, especially factors such as socioeconomic status or other public health policies. Due to data limitations, our results might either overestimate or underestimate the true effects, thereby affecting the generalizability of our findings. Fourth, we applied first-order differencing to our variables of interest, and this restricted us to predict a long-term consequence as it focuses on short-term changes after differencing.

## Conclusion

COVID-19 significantly disrupted the balance between blood supply and demand, particularly affecting blood types A and O the most. However, these imbalances gradually recovered over time, demonstrating the resilience of the blood supply chain through proactive strategies. Furthermore, our study highlighted the positive effect of group donations on the local economic performance. The findings underscore the important role that group donations play in blood collection and the effectiveness of proactive strategies—collective community initiatives and mobilization—in mitigating the adverse effects of public health crises on the healthcare system and economy.

## Data Availability

The datasets presented in this study can be found in online repositories. The names of the repository/repositories and accession number(s) can be found in the article/Supplementary material.

## References

[1] China’s response to Covid-19|The BMJ. Available online at: https://www.bmj.com/how-china-responded-to-covid-19 (Accessed October 6, 2024).

[2] Wang G Chen W Jin X Chen P. Description of COVID-19 cases along with the measures taken on prevention and control in Zhejiang, China. J Med Virol. 92:1948–55. doi: 10.1002/jmv.25906, 32311151 PMC7264658

[3] Sun Y Wang WY. Governing with health code: standardising China’s data network systems during COVID-19. Policy Internet. 14:673–89. doi: 10.1002/poi3.292 PMC9088356 35573035

[4] Sun S Xie Z Yu K Jiang B Zheng S Pan X. COVID-19 and healthcare system in China: challenges and progression for a sustainable future. Glob Health. (2021) 17:14. doi: 10.1186/s12992-021-00665-9, 33478558 PMC7819629

[5] Loua A Kasilo OMJ Nikiema JB Sougou AS Kniazkov S Annan EA. Impact of the COVID-19 pandemic on blood supply and demand in the WHO African region. Vox Sang. (2021) 116:774–84. doi: 10.1111/vox.13071, 33529421 PMC8014179

[6] Guidelines for prevention and control of SARS-CoV-2 infection in blood centers (in Chinese). Available online at: https://www.nhc.gov.cn/ylyjs/gzdt/202212/eb9dce4b02c147099b11756d11bbf227/files/1734003020149_84710.pdf (Accessed January 12, 2026).

[7] Blood types. Available online at: https://www.redcrossblood.org/donate-blood/blood-types.html (Accessed October 10, 2024).

[8] Gammon RR Rosenbaum L Cooke R Friedman M Rockwood L Nichols T . Maintaining adequate donations and a sustainable blood supply: lessons learned. Transfusion (Paris). (2021) 61:294–302. doi: 10.1111/trf.16145, 33206404 PMC7753343

[9] Gao D Li H Wang K. The development of a legal framework for blood donation and blood safety in China over 24 years. BMC Health Serv Res. (2020) 20:1099. doi: 10.1186/s12913-020-05944-6, 33256716 PMC7702669

[10] 20 Questions about voluntary blood donation (in Chinese). Available online at: https://xyy.cupl.edu.cn/info/1010/1253.htm (Accessed October 10, 2024).

[11] Zhejiang provincial blood center. Available online at: https://www.zjb.org.cn/index.php?s=/Home/News/search.html&title=%E5%9B%A2%E9%98%9F%E7%8C%AE%E8%A1%80 (Accessed October 10, 2024).

[12] McKibbin W Fernando R. The global economic impacts of the COVID-19 pandemic. Econ Model. (2023) 129:106551. doi: 10.1016/j.econmod.2023.106551

[13] Mandel A Veetil V. The economic cost of COVID lockdowns: an out-of-equilibrium analysis. Econ Disasters Clim Change. (2020) 4:431–51. doi: 10.1007/s41885-020-00066-z, 32838118 PMC7304379

[14] Hakami NY Al-Sulami AJ Alhazmi WA Qadah TH Bawazir WM Hamadi AY . Impact of COVID-19 on blood donation and supply: a multicenter cross-sectional study from Saudi Arabia. Biomed Res Int. (2022) 2022:1474426. doi: 10.1155/2022/1474426 35036427 PMC8756159

[15] Al-Riyami AZ Abdella YE Badawi MA Panchatcharam SM Ghaleb Y Maghsudlu M . The impact of COVID-19 pandemic on blood supplies and transfusion services in eastern Mediterranean region. Transfus Clin Biol. (2021) 28:16–24. doi: 10.1016/j.tracli.2020.11.002 33276150 PMC7706593

[16] Junfen L Mengna W. Analysis of epidemiological characteristics of COVID-19 cases in Zhejiang Province. Prevent Med (in Chinese). (2020) 32:217–22.

[17] Bernal JL Cummins S Gasparrini A. Interrupted time series regression for the evaluation of public health interventions: a tutorial. Int J Epidemiol. (2016) 46:348. doi: 10.1093/ije/dyw098 PMC5407170 27283160

[18] Linden A. Conducting interrupted time-series analysis for single- and multiple-group comparisons. Stata J. (2015) 15:480–500. doi: 10.1177/1536867x1501500208

[19] Turner SL Karahalios A Forbes AB Taljaard M Grimshaw JM McKenzie JE. Comparison of six statistical methods for interrupted time series studies: empirical evaluation of 190 published series. BMC Med Res Methodol. (2021) 21:134. doi: 10.1186/s12874-021-01306-w, 34174809 PMC8235830

[20] Loua A Kasilo OMJ Nikiema JB Sougou AS Kniazkov S Annan EA. Impact of the COVID-19 pandemic on blood supply and demand in the WHO African region. Vox Sang. (2021) 116:774–84. doi: 10.1111/vox.13071, 33529421 PMC8014179

[21] Saillant NN Kornblith LZ Moore H Barrett C Schreiber MA Cotton BA . The national blood shortage—an impetus for change. Ann Surg. (2022) 275:641–3. doi: 10.1097/SLA.0000000000005393, 35081570 PMC9055632

[22] Vasconcelos FT Faddy HM Merollini KMD Flower RLP Dean MM Viennet E. Impact of natural disasters and pandemics on blood supply: a systematic review. Health Sci Rev. (2023) 7:100087. doi: 10.1016/j.hsr.2023.100087

[23] Hultström M Persson B Eriksson O Lipcsey M Frithiof R Nilsson B. Blood type A associates with critical COVID-19 and death in a Swedish cohort. Crit Care. (2020) 24:496. doi: 10.1186/s13054-020-03223-8, 32787887 PMC7422469

[24] Zietz M Zucker J Tatonetti NP. Associations between blood type and COVID-19 infection, intubation, and death. Nat Commun. (2020) 11:5761. doi: 10.1038/s41467-020-19623-x, 33188185 PMC7666188

[25] Wang F Xu Z Lv K Fei J Yang H Ou Z . Analysis of blood utilisation efficiency driven by clinical management and hospital heterogeneity. Front Public Health. (2025) 13:1668449. doi: 10.3389/fpubh.2025.1668449 41080868 PMC12507902

[26] Sun Y Wang L Niu J Ma T Xing L Song A . Distribution characteristics of ABO blood groups in China. Heliyon. (2022) 8:e10568. doi: 10.1016/j.heliyon.2022.e10568 36119853 PMC9479019

[27] Zhao J Yang Y Huang H Li D Gu D Lu X . Relationship between the ABO blood group and the coronavirus disease 2019 (COVID-19) susceptibility. Clin Infect Dis Off Publ Infect Dis Soc Am. (2021) 73:328–31. doi: 10.1093/cid/ciaa1150 PMC7454371 32750119

[28] Kim Y Latz CA DeCarlo CS Lee S Png CYM Kibrik P . Relationship between blood type and outcomes following COVID-19 infection. Semin Vasc Surg. (2021) 34:125–31. doi: 10.1053/j.semvascsurg.2021.05.005, 34642032 PMC8286549

[29] With a surge in positive cases and a shortage of blood supply, how can blood provision be ensured (in Chinese). Available online at: https://export.shobserver.com/baijiahao/html/565071.html (Accessed January 14, 2025).

[30] Blood donors across many regions in Zhejiang contribute to the fight against the pandemic (in Chinese). Available online at: https://baijiahao.baidu.com/s?id=1662686270622797366&wfr=spider&for=pc (Accessed January 14, 2025).

[31] Yu S Yao Y EICA group. The influence of the COVID-19 pandemic on blood donation and supply in China. Transfus Med. (2024) 34:124–35. doi: 10.1111/tme.13020 38151821

[32] Wang Y Han W Pan L Wang C Liu Y Hu W . Impact of COVID-19 on blood centres in Zhejiang province China. Vox Sang. (2020) 115:502–6. doi: 10.1111/vox.12931, 32347566 PMC7267653

[33] Gasparovic Babic S Krsek A Baticic L. Voluntary blood donation in modern healthcare: trends, challenges, and opportunities. Epidemiologia. (2024) 5:770–84. doi: 10.3390/epidemiologia5040052, 39727424 PMC11675880

[34] Promote voluntary group blood donation and welcome registrations from enterprises and public institutions (in Chinese). Available online at: http://www.tjbc.org.cn/system/2016/05/30/012250991.shtml (Accessed January 16, 2025).

[35] Kumar A Kumari S Saroj U Verma A Kiran KA Prasad MK . Impact of the COVID-19 pandemic on blood donation patterns: a systematic review and meta-analysis. Cureus. 15:e43384. doi: 10.7759/cureus.43384, 37700994 PMC10495075

[36] Sun P Zhu L Ma L Li C Wang Z Zhang R . Blood service in a region of China’s Qinghai–Tibetan plateau. Healthcare. (2023) 11:1944. doi: 10.3390/healthcare11131944, 37444778 PMC10341248

[37] Chen J Ou-Yang J Xie G Liang H Fan Y Gao R . Problems and challenges: development of blood transfusion services in mainland China within the context of health-care system reform. Transfus Med. (2019) 29:253–61. doi: 10.1111/tme.12618, 31359545

[38] Ping H Xing N. Blood shortages and donation in China. Lancet. (2016) 387:1905–6. doi: 10.1016/S0140-6736(16)30417-2, 27203650

[39] Wang Z Wang H. Exploring blood donation challenges and mobilization mechanisms in North China during the COVID-19 pandemic: a qualitative study. Risk Manag Healthc Policy. (2022) 15:1593–605. doi: 10.2147/RMHP.S372945, 36061880 PMC9433754

[40] Siu JY Chan EA Li AS Lee YM. Barriers to engaging in blood donation during the COVID-19 pandemic among nondonors and lapsed donors in a Chinese community: a critical medical anthropology perspective. Health Expect. (2025) 28:e70236. doi: 10.1111/hex.70236, 40176358 PMC11965271

